# Yeast [FeFe]-hydrogenase-like protein Nar1 binds a [2Fe–2S] cluster

**DOI:** 10.1039/d5sc04860e

**Published:** 2025-11-10

**Authors:** Joseph J. Braymer, Lukas Knauer, Jason C. Crack, Jonathan Oltmanns, Melanie Heghmanns, Jéssica C. Soares, Nick E. Le Brun, Volker Schünemann, Müge Kasanmascheff

**Affiliations:** a Fachbereich Medizin, Institut für Zytobiologie und Zytopathologie & Zentrum für Synthetische Mikrobiologie (Synmikro), Philipps-Universität Marburg Karl-von-Frisch-Str. 14 35032 Marburg Germany; b Bundesanstalt für Materialforschung und -prüfung (BAM) 12205 Berlin Germany joseph.braymer@bam.de; c Fachbereich Physik, Rheinland-Pfälzische Technische Universität Kaiserslautern-Landau Erwin-Schrödinger-Str. 46 D-67663 Kaiserslautern Germany; d Fachbereich Chemie, Rheinland-Pfälzische Technische Universität Kaiserslautern-Landau Erwin-Schrödinger-Str. 52 D-67663 Kaiserslautern Germany; e Centre for Molecular and Structural Biochemistry, School of Chemistry, Pharmacy, and Pharmacology, University of East Anglia Norwich Research Park Norwich NR4 7TJ UK; f Fakultät für Chemie und Chemische Biologie, Technische Universität Dortmund Otto-Hahn-Straße 6 44227 Dortmund Germany

## Abstract

Nar1 is an essential eukaryotic protein proposed to function as an iron–sulphur (Fe/S) cluster trafficking factor in the cytosolic iron–sulphur protein assembly (CIA) machinery. However, such a role has remained unclear due to difficulties in purifying adequate amounts of cofactor-bound protein. The [FeFe]-hydrogenase-like protein has two conserved binding sites for [4Fe–4S] clusters but does not show hydrogenase activity *in vivo* due to the lack of an active site [2Fe]_H_ cofactor. Here, we report a new preparation procedure for Nar1 that facilitated studies by UV-vis, EPR, and Mössbauer spectroscopies, along with native mass spectrometry. Nar1 recombinantly produced in *E. coli* contained a [4Fe–4S] cluster, bound presumably at site 1, along with an unexpected [2Fe–2S] cluster bound at an unknown site. Fe/S reconstitution reactions installed a second [4Fe–4S] cluster at site 2, leading to protein with up to three Fe/S cofactors. It is proposed that the [2Fe–2S] cluster occupies a cavity in Nar1 that is filled by the [2Fe]_H_ cofactor in [FeFe]-hydrogenases. Strikingly, two of the Fe/S clusters were rapidly destroyed by molecular oxygen, linking Nar1 oxygen sensitivity *in vitro* to phenotypes observed previously *in vivo*. Our biochemical results, therefore, validate a direct link between cellular oxygen concentrations and the functioning of the CIA pathway. These advances also now allow for the pursuit of *in vitro* Fe/S cluster transfer assays, which will shed light on Fe/S trafficking and insertion by CIA components.

## Abstract 2

Nar1 is an essential iron–sulphur protein biogenesis component in eukaryotes that can bind not only two [4Fe–4S] clusters but also a newly identified [2Fe–2S] cluster.

## Graphical abstract

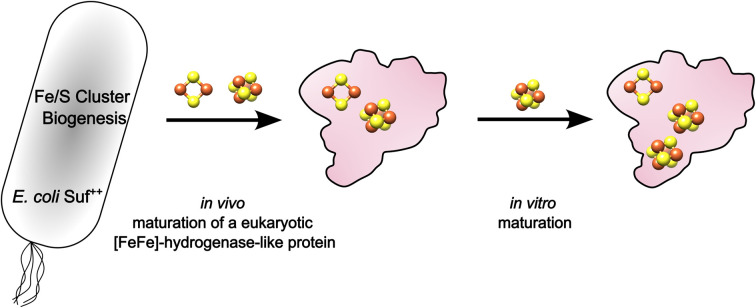

## Introduction

The generation, trafficking, and insertion of [4Fe–4S] clusters into cytosolic and nuclear [4Fe–4S] proteins is a fundamental reaction required for the proper maturation of essential DNA/RNA processing enzymes, amongst many others.^1,2^ These functions are carried out by the cytosolic iron–sulphur (Fe/S) protein assembly (CIA) machinery in eukaryotes, which can involve up to 13 proteins and is also fully dependent on the mitochondrial Fe/S cluster assembly (ISC) system.^3–6^ In the model of [4Fe–4S] cluster biogenesis in the cytosol,^3^ tetranuclear clusters are generated on the yeast scaffolding proteins Cfd1 and Nbp35 *via* the assistance of reducing power from the electron transfer complex involving NADPH, Tah18, and Dre2.^7,8^ The generated [4Fe–4S] clusters are then trafficked to Nar1,^9–11^ which connects further to Cia1, Cia2, and Mms19 of the CIA targeting complex (CTC) that is responsible for the final insertion of Fe/S clusters into target proteins.^12,13^

**Fig. 1:**
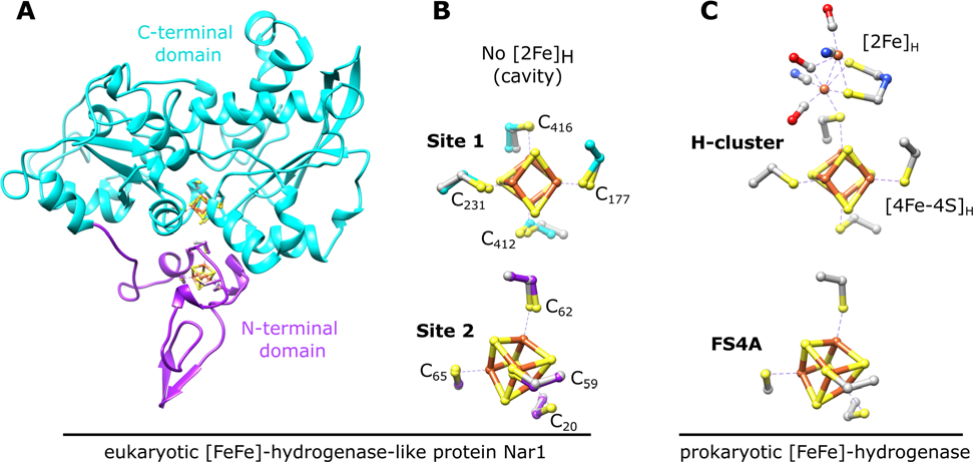
Nar1 is predicted to bind two [4Fe–4S] clusters based on homology with prokaryotic [FeFe]-hydrogenases. (A) Predicted protein structure of Nar1 showing the N-terminal ferredoxin-like domain (magenta) and the C-terminal domain (cyan). The yeast Nar1 sequence was threaded through the [FeFe]-hydrogenase from *Clostridium pasteurianum* (PDB 1FEH) using SwissModel^24^ and the [4Fe–4S] clusters from 1FEH were overlayed onto the modelled Nar1 structure. (B) Cysteine residues of the two predicted [4Fe–4S] cluster binding sites of Nar1 are labelled and shown as in (A). Corresponding coordinating cysteines from 1FEH are shown in grey. (C) The H-cluster, composed of [2Fe]_H_ and [4Fe–4S]_H_ that are held in place by a conserved bridging cysteine, and the proximal [4Fe–4S] cluster (FS4A) of [FeFe]-hydrogenases are shown (PDB 4XDC).

Nar1 is a structural [FeFe]-hydrogenase homolog that lacks hydrogenase activity and may be both a CIA component and a potential target of the CIA machinery, as it contains two conserved [4Fe–4S] binding sites (Fig. 1) along with a C-terminal tryptophan for targeting to the CTC (Fig. S1).^9,10,14^ Site 1 in the C-terminal domain is homologous to the [4Fe–4S]_H_ cluster binding site of the H-cluster in [FeFe]-hydrogenases (Fig. 1). A complete H-cluster including the unique 2Fe cluster, [2Fe]_H_, is not found in eukaryotic yeast and human Nar1 homologs as the maturation proteins are lacking.^10^ Site 2 of Nar1 contains four conserved cysteine residues comparable to the FS4A cluster binding site in [FeFe]-hydrogenases (Fig. 1 and S1).^15^ Previous attempts to purify Nar1 from several organisms have yielded protein with predominately only one [4Fe–4S] cluster in site 1, precluding a thorough biochemical and biophysical analysis of the protein.^9,11,16,17^ While other CIA components have been isolated and biophysically characterized,^12,18–21^ the inability to produce mature Nar1 has also prevented the development of *in vitro* CIA pathway assays of the type established for the early and late ISC machineries.^22,23^

## Results and discussion

### Isolation of Nar1 with elevated iron and sulphur content

In an attempt to produce mature Nar1, an *E. coli* host strain with a functional SUF machinery (Suf^++^)^25^ was utilized to express N-terminally His-tagged and C-terminally Strep-tagged Nar1 from *Saccharomyces cerevisiae* (SI Methods). Recombinant Nar1 was isolated and purified under anaerobic conditions as a predominately monomeric, brown-coloured protein solution with UV-vis absorption features at 320 and 420 nm indicative of [2Fe–2S] and/or [4Fe–4S] clusters (Fig. S2).^26^ Native mass spectrometry confirmed a monomeric, folded state of Nar1 (Fig. S3). The iron and acid labile sulphide content per mole of protein was 2.9 ± 0.1 and 4.6 ± 0.2, respectively. In an attempt to bolster the Fe/S content, chemical reconstitution with iron and sulphide was employed.^26^ Notably, increased amounts of iron and sulphur could be detected in the monomeric, reconstituted protein, 7.8 ± 0.2 and 10.8 ± 0.7, respectively. Consistent with increased iron/sulphur, the UV-vis spectrum showed an increase in the 320 and 420 nm signals (Fig. S2B).

### Site specific ^57^Fe labelling of Fe/S clusters

**Fig. 2:**
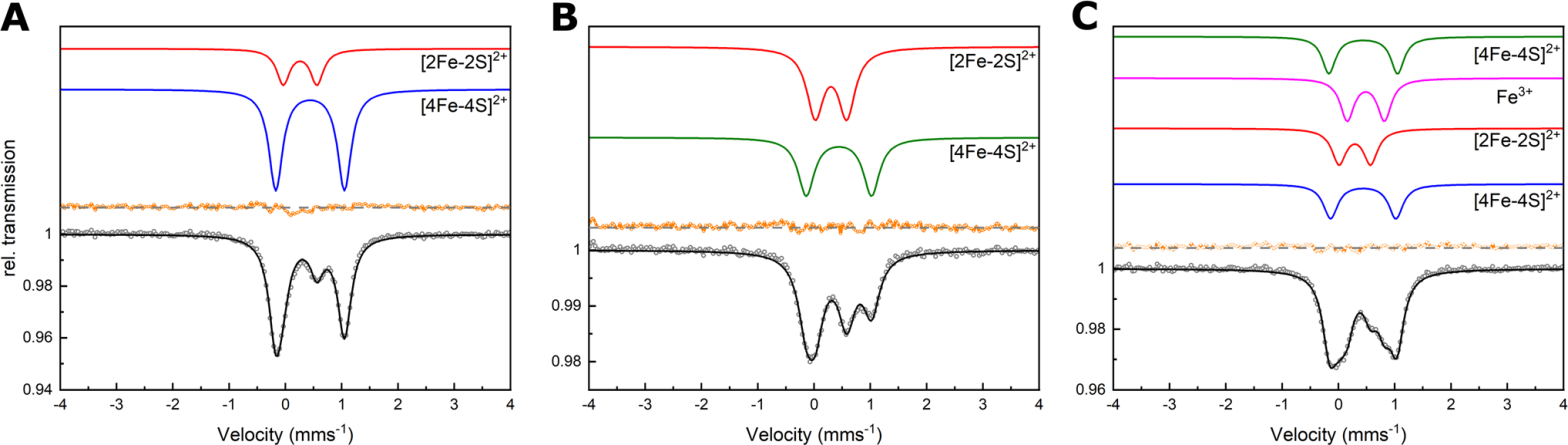
Mössbauer spectroscopy indicated the presence of [2Fe–2S] and [4Fe–4S] clusters bound to Nar1. (A) Spectra of as-isolated Nar1 with ^57^Fe labelling, (B) Nar1 with ^56^Fe/^57^Fe labelling, and (C) Nar1 with ^57^Fe/^57^Fe labelling. Experimental data are shown as open circles with corresponding experimental error. Simulations are shown for the individually assigned components (in colour) and of the experimental data combining the individual components (black). Summed simulations subtracted from the experimental data are shown as residual plots (orange). Spectra were collected at 77 K with protein concentrations of 520 µM (A), 230 µM (B), and 180 µM (C). For parameters see Table 1.

To characterize Nar1 in greater detail, Mössbauer spectroscopy was employed at various temperatures and magnetic fields using protein samples labelled with ^57^Fe. Nar1 was first prepared with ^57^Fe in the expression medium and analysed as isolated under anaerobic conditions (^57^Fe labelling). The experimental data were simulated with two components. Component 1 (Fig. 2A, blue line, Table 1) had an isomer shift (*δ*) of 0.44 mm s^−1^ and a quadrupole splitting (Δ*E*_Q_) of 1.22 mm s^−1^. These parameters are characteristic of [4Fe–4S]^2+^ clusters coordinated by cysteine ligands.^27,28^ Unexpectedly, component 2 was simulated with *δ* = 0.26 mm s^−1^ and Δ*E*_Q_ = 0.60 mm s^−1^, consistent with parameters for [2Fe–2S]^2+^ clusters (Fig. 2A, red line and Table 1).^29^ Both components were sub-stoichiometric compared to monomeric protein suggesting that each site was only partially filled (Table 1). Field-dependent Mössbauer spectroscopy at 4.2 K displayed magnetic hyperfine splitting, attributable solely to the external field, and unambiguously confirmed the presence of [2Fe–2S]^2+^ and [4Fe–4S]^2+^ clusters (Fig. S4A, B and Table S1).

**Table 1:** Parameters as obtained from the analysis of the Mössbauer spectra shown in Fig. 2

Assignment	^57^Fe labelling (2.0 mM Fe)^a^		^56^Fe/^57^Fe labelling (1.1 mM Fe)		^57^Fe labelling (1.9 mM Fe)			
	[4Fe–4S]^2+^	[2Fe–2S]^2+^	[4Fe–4S]^2+^	[2Fe–2S]^2+^	[4Fe–4S]^2+^	[4Fe–4S]^2+^	[2Fe–2S]^2+^	Fe^3+^
*δ* (mms^−1^)	0.44	0.26	0.44	0.30	0.44	0.44	0.29	0.49
Δ*E*_Q_ (mms^−1^)	1.22	0.60	1.16	0.56	1.22	1.16	0.56	0.66
*Γ* (mms^−1^)	0.29	0.28	0.35	0.36	0.29	0.31	0.31	0.30
Area (%)	75	25	45	55	24	24	24	28
Component per protein	0.72	0.48	0.51	1.3	0.64	0.64	1.3	3.0

a Iron concentrations were determined by the colorimetric Ferene assay (see SI Methods).

Next, ^57^Fe was utilized in the chemical reconstitution reaction using as-isolated protein expressed in medium containing ^56^Fe (^56^Fe/^57^Fe labelling). Under these conditions, the experimental data could again be simulated with contributions from [4Fe–4S]^2+^ and [2Fe–2S]^2+^ clusters with slightly altered parameters as compared to the as-isolated protein (Fig. 2B, S4C, D and Table 1). Simulation of the ^56^Fe/^57^Fe labelling data with parameters of the as-isolated [4Fe–4S]^2+^ cluster resulted in a slightly worse simulation (Fig. S5A and Table S2), which suggested the presence of a [4Fe–4S]^2+^ cluster in an electronic environment somewhat distinct from that of the as-isolated sample. Small differences in isomer shift and quadruple splitting for the [2Fe–2S]^2+^ cluster suggested either the presence of the same [2Fe–2S] cluster as in the as-isolated sample, but in a slightly different electronic environment due to conformational changes caused by an additional [4Fe–4S] cluster, or a [2Fe–2S] cluster at a new binding site. In the former case, the presence of the same [2Fe–2S] cluster as in the as-isolated sample could result from the facile exchange of ^56^Fe for ^57^Fe at a solvent accessible [2Fe–2S] cluster.^30^ Compared to monomeric protein, the [2Fe–2S] cluster component was stoichiometric whereas the signals for the new [4Fe–4S] cluster were sub-stoichiometric (Table 1).

Lastly, the as-isolated sample prepared with ^57^Fe in the medium was also reconstituted in the presence of ^57^Fe so as to label all possible Fe/S clusters in Nar1 (^57^Fe/^57^Fe labelling, Fig. 2C, S4E and F). The experimental data were simulated by two [4Fe–4S]^2+^ species with the same parameters as in the ^57^Fe and ^56^Fe/^57^Fe labelling schemes, in addition to a [2Fe–2S] species that closely matched that observed following ^56^Fe/Fe^57^ labelling (Fig. 2C and Table 1). Notably, the two [4Fe–4S] clusters contributed equally to the Mössbauer spectrum (Table 1). In addition to Fe/S clusters, another component was assigned as Fe^3+^ stemming from either unusual Fe isotope exchange of Fe/S clusters or the reconstitution reaction (Fig. 2C, S4, S5B and S6). Therefore, Mössbauer spectroscopy traced the presence of at least two and possibly three distinct Fe/S clusters inserted either *in vivo* or *in vitro*.

Samples from ^57^Fe and ^57^Fe/^57^Fe labelling were further treated with sodium dithionite (DT) to characterize the Fe/S clusters in the reduced state. For ^57^Fe labelling, a split signal consistent with a mixed valent [4Fe–4S]^+^ cluster was simulated (*δ*_1_ = 0.42 mm s^−1^ and Δ*E*_Q1_ = 1.15 mm s^−1^; *δ*_2_ = 0.62 mm s^−1^ and Δ*E*_Q2_ = 1.18 mm s^−1^, Fig. S7A, B and Table S3). A weak signal at *δ* = 0.72 mm s^−1^ and Δ*E*_Q_ = 3.25 mm s^−1^ corresponded to Fe^2+^ at a tetrahedral site, but not a [2Fe–2S]^+^ cluster, as the signal remained unchanged upon lowering the temperature from 200 to 86 K (Fig. S7A–D).^28^ In a similar manner, reduction of the ^57^Fe/^57^Fe labelled sample resulted in a mixed-valent [4Fe–4S]^+^ species along with multiple mononuclear Fe^2+^ species (Fig. S7E, F and Table S3). One of the Fe^2+^ species was within the range of tetrahedral Fe^2+^ sites (*δ* = 0.86 mm s^−1^ and Δ*E*_Q_ = 2.87 mm s^−1^) while the other two are assigned as non-specific octahedral Fe^2+^ species with *δ* > 1.2 mm s^−1^ (Fig, S7E and Table S3). These results suggested that in both samples, [2Fe–2S] clusters are either unstable in the reduced state resulting in the generation of mononuclear Fe^2+^, and/or are converted to the diferrous state [2Fe–2S]^0^.^31^ Furthermore, for as-isolated Nar1 (^57^Fe labelling), reduction with DT scrambled the Fe/S content, as evidenced by an increase in the spectral contribution of [4Fe–4S] content from 75% to 90% (Table 1 and S3).^32^ In the reconstituted sample (^57^Fe/^57^Fe labelling), a small increase in [4Fe–4S] content was observed and an increase in non-specific iron was also detected. Therefore, whereas [4Fe–4S]^+^ clusters in Nar1 are stable, [2Fe–2S]^+^ clusters cannot be detected by Mössbauer due to their instability. Reduction of labile [2Fe–2S]^2+^ clusters in monothiol glutaredoxins also results in the loss of the Fe/S cluster.^33^

### Magnetic exchange coupling between Fe/S clusters

**Fig. 3:**
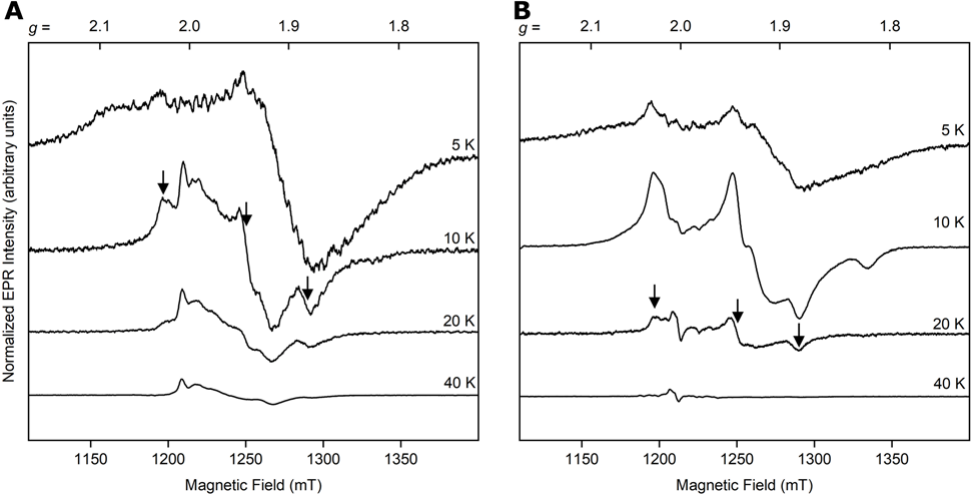
Temperature-dependent behaviour of as-isolated (A, 930 µM) and reconstituted (B, 480 µM) Nar1 reduced with 10 equiv. of DT observed by pulsed Q-band EPR (34 GHz). At 10 K in (A), a signal for a [4Fe–4S]^+^ cluster is apparent, which is comparable to the signal at 20 K in (B) for reconstituted protein (marked by arrows). Refer to Fig. S8 for the contributions of [2Fe–2S]^+^ cluster species in as-isolated protein at 40 K in (A). The radical signal at 40 K in (B) likely stems from DT. All spectra are corrected for concentration.

To gain more insight into the reduced Fe/S content of as-isolated and reconstituted Nar1, we utilized variable-temperature EPR spectroscopy at 34 GHz/1.2 mT (Q-band). The Q-band spectrum of as-isolated Nar1 reduced with DT showed a broad and complex signal at 10 K (Fig. 3A). A rhombic-like signal at 10 K broadened into the baseline as the temperature was raised to 20 and 40 K. This is consistent with the presence of at least one low spin *S* = ½ [4Fe–4S]^+^ cluster, which is not detectable above 20 K due to fast relaxation (Fig. 3A).^34,35^ Notably, at 5 K the spectrum broadened significantly, indicative of magnetic exchange coupling between fast-relaxing Fe/S clusters. Spectra acquired at higher temperatures of 40 and 60 K showed some signals remaining (Fig. 3A bottom trace and S8). These weaker signals corresponded to slow-relaxing low spin *S* = ½ [2Fe–2S]^+^ clusters^34,35^ and are more complicated than a single rhombic [2Fe–2S] cluster signature that is found, for example, in [2Fe–2S] ferredoxins.^36,37^ The Nar1 spectrum is reminiscent of multiple [2Fe–2S]^+^ components detected in bioB and anamorsin,^38,39^ which suggested multiple [2Fe–2S] species (Fig. S8). As a [2Fe–2S]^+^ cluster had not been resolved in the Mössbauer experiment, it is suspected that the [2Fe–2S]^+^ cluster contribution observed by EPR is a minor component stemming either from an intermediate during degradation of Fe/S clusters^33^ or from the assembly of a new [2Fe–2S] cluster in a different site under the reducing conditions.^32^

Next, we investigated the reconstituted protein by EPR. In agreement with Mössbauer, no signals for [2Fe–2S]^+^ species were observed at higher temperatures (Fig. 3B, bottom trace). Upon lowering the temperature to 20 K, a rhombic signature appeared with approximate *g* values of *g*_1_ ≈ 2.028, *g*_2_ ≈ 1.941, and *g*_3_ ≈ 1.882, similar to that observed with the as-isolated sample (Fig. 3A and B). While these *g*-values are in the range of [4Fe–4S]^+^ clusters, they are different from axial and rhombic EPR signals observed previously for Nar1 and [FeFe]-hydrogenases.^10,11,16,40,41^ Microheterogeneity in and around [4Fe–4S]^+^ clusters has been demonstrated to influence *g*-values.^41^ At 10 K, the spectrum broadened with additional features becoming apparent (*e.g.*, at *g* ≈ 1.819) due most likely to at least one additional, faster-relaxing [4Fe–4S]^+^ cluster. The spectrum at 5 K was further broadened, again suggestive of magnetic coupling of [4Fe–4S]^+^ clusters (Fig. 3B, top trace).^40,42–44^ We note that [2Fe–2S]^+^ and [4Fe–4S]^+^ clusters were not present in the non-reduced samples, only minor amounts of [3Fe–4S]^+^ clusters were apparent (Fig. S9). Overall, EPR analysis is consistent with the presence of [4Fe–4S] clusters in Nar1 that are magnetically coupled to other species. As reduction largely destroyed the [2Fe–2S] clusters and redistributed Fe content in both samples, it is expected that the signal broadening observed at low temperature stemmed from [4Fe–4S] clusters in sites 1 and 2.

### Native MS confirms double- and triple-occupied Nar1 states

**Fig. 4:**
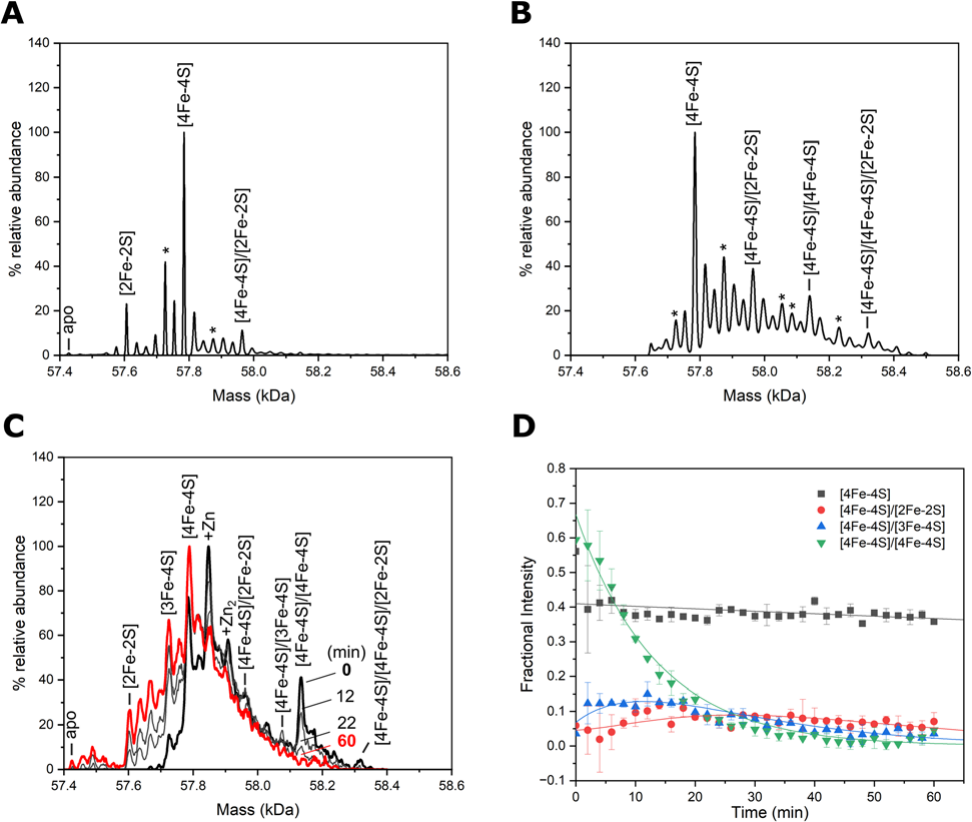
Native mass spectrometry of Nar1. (A) As-isolated Nar1 binds up to two Fe/S clusters, with a [4Fe–4S] cluster predicted in site 1 and [2Fe–2S] cluster at an unknown site, in addition to singly occupied Fe/S species. (B) Chemically reconstituted Nar1 binds a second [4Fe–4S] cluster most likely in site 2, resulting in [4Fe–4S]/[4Fe–4S] and [4Fe–4S]/[4Fe–4S]/[2Fe–2S] species. Additional species are observed due to cluster breakdown (labelled with *) or sulphur adducts (see Fig. S10 and Table S4). (C) Spectra of enzymatically reconstituted Nar1 before (black line), and after exposure to dissolved atmospheric oxygen (60 min exposure; red line), measured on a lower resolution instrument set up for kinetics. Intervening spectra (grey lines), recorded at 12- and 22-min exposure show rapid loss of one of the [4Fe–4S] clusters. Cluster types are labelled in A–C based on molecular masses (Table S4) and analysis by spectroscopic methods. (D) Temporal analysis of the [4Fe–4S] clusters in response to O_2_. One of the clusters in [4Fe–4S]/[4Fe–4S]-Nar1 rapidly degrades to unstable [3Fe–4S] and [2Fe–2S] clusters. We note that the [4Fe–4S]/[4Fe–4S]/[2Fe–2S]-Nar1 species under the conditions in (B) is a Li^+^ adduct (see Table S4) and in (C) was too low in intensity to provide reliable kinetics. See Fig. S11 for the temporal analysis of additional cluster degradation species.

Finally, we used native mass spectrometry coupled with ion mobility (IMS-MS) to study the various species of Nar1 in the gas phase.^45^ Analysing the folded species (Fig. S3) showed Nar1 with various cofactor occupancies in the as-isolated protein (Fig. 4). The highest abundance peak at 57 784 Da corresponded to Nar1 containing one [4Fe–4S] cluster (theoretical mass 57 785 Da), but protein bound to one [4Fe–4S] and one [2Fe–2S] cluster was also observed at a mass of 57 964 Da (Fig. 4A and Table S4). Protein containing single [2Fe–2S] and [3Fe–4S] clusters were also observed in addition to a weak signal for apo protein. Measurement of the chemically reconstituted sample revealed additional peaks at 58 139 and 58 321 Da corresponding to [4Fe–4S]/[4Fe–4S]-Nar1 and [4Fe–4S]/[4Fe–4S]/[2Fe–2S]-Nar1, respectively (Fig. 4B and Table S4).

These observations have three important implications for cofactors binding to Nar1: (1) verification of a double-occupied cofactor state of as-isolated protein ([2Fe–2S] and [4Fe–4S] clusters binding simultaneously), (2) two [4Fe–4S] clusters binding to Nar1 in the reconstituted state, and (3) first occurrence of a third site being occupied by a [2Fe–2S] cluster, which was hinted at in the Mössbauer spectra. Additionally, disassembly of the [2Fe–2S] cluster leading to [4Fe–4S]/[Fe–S]-Nar1 and [4Fe–4S]/[4Fe–4S]/[Fe–S]-Nar1 adducts was observed by MS supporting the observation by Mössbauer spectroscopy that the [2Fe–2S] cluster originally bound to Nar1 is unstable to reductive conditions (Fig. S10 and Table S4). Both [4Fe–4S]/[4Fe–4S]-Nar1 and [4Fe–4S]/[4Fe–4S]/[2Fe–2S]-Nar1 could also be independently generated using a semi-biosynthetic reconstitution of Fe/S clusters employing the desulphurase NifS (Fig. 4C).^46^ Strikingly, one of the [4Fe–4S] clusters in these states was highly prone to degradation by molecular oxygen under the experimental conditions (*t*_1/2_ ≈ 9 min) whereas the singly occupied [4Fe–4S]-Nar1 species showed much greater stability (Fig. 4C and D). The latter experiment indicated that the presence of Nar1 associated with only [2Fe–2S] or [3Fe–4S] clusters stemmed from the decay of [4Fe–4S] clusters (Fig. 4, S10 and 11).

### Model of Fe/S cluster insertion into Nar1

**Fig. 5:**
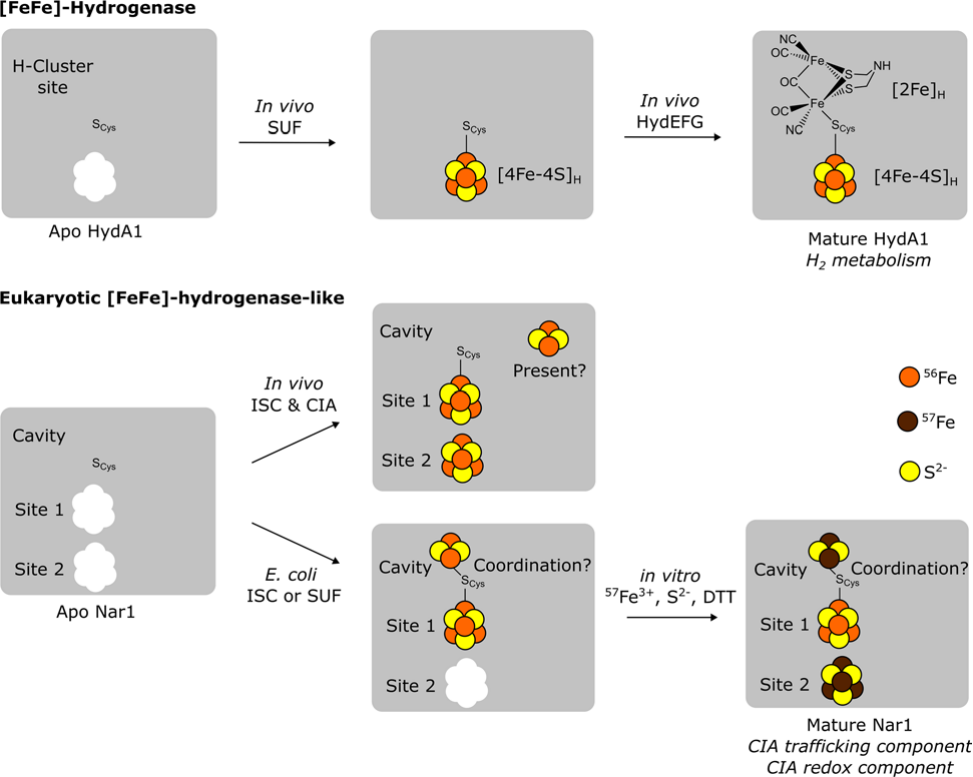
Models for the loading of cofactors into the homologous proteins [FeFe]-hydrogenase HydA1 (ref. 55) (top) and Nar1 (bottom). In [FeFe]-hydrogenases, a bridging cysteine (S_cys_) coordinates both the [4Fe–4S]_H_ cluster and the unique [2Fe]_H_ cluster. In addition to a [4Fe–4S] cluster, Nar1 purified from *E. coli* is capable of binding at least one [2Fe–2S] cluster, which is hypothesized to be coordinated in an unknown fashion in the cavity of Nar1 that is analogous to the [2Fe]_H_ binding site (S_cys_ = Cys_416_ in Nar1). *In vitro* chemical reconstitution of isolated protein maintains a [2Fe–2S] cluster and adds a second [4Fe–4S] cluster in site 2, a reaction that can be traced by MS and by isotopic labelling in Mössbauer experiments.

Altogether, the biophysical data presented here provides new insight into the insertion of Fe/S clusters into the essential yeast protein Nar1 (Fig. 5). As the as-isolated protein contained both [2Fe–2S] and [4Fe–4S] clusters, it is proposed that recombinantly expressed Nar1 purifies from the *E. coli* Suf^++^ strain predominately with a [4Fe–4S] cluster at Fe/S site 1 as in *Chlamydomonas reinhardtii* HydA1 (Fig. 5)^9,11,16,41,47^ and at least one [2Fe–2S] cluster. The latter Fe/S cluster may have been critical for the chemical reconstitution of a second [4Fe–4S] cluster at Fe/S site 2. Iron and sulphide determinations along with Mössbauer spectroscopy indicated that in both as-isolated and reconstituted protein, not all sites are fully occupied, potentially due to either protein quality (conformations of apo protein incapable of cofactor binding) or lability of Fe/S clusters, as observed here and previously.^9,47^ Yet, the spectroscopic and spectrometric data on reconstituted samples presented here clearly demonstrated the binding of a second [4Fe–4S] cluster in site 2 of Nar1. Although the Suf^++^ strain facilitated the insertion of at least one [2Fe–2S] cluster into Nar1, our data, in addition to previous studies,^9–11,16^ suggest that the [4Fe–4S] cluster at Fe/S site 2 cannot be readily inserted *in vivo* by prokaryotic ISC or SUF machineries.^48^ The dedicated loading of the N-terminal [4Fe–4S] cluster *in vivo*^9,10^ therefore would be hypothesized to only be accomplished by the essential CIA scaffolding complexes involving Nbp35 or Cfd1/Nbp35 in eukaryotes (Fig. 5).^7^ This assumption would be consistent with the essentiality of Nbp35 in eukaryotes and its retention in the minimal CIA system of *Monocercomonoides exilis*.^3,49^

*In vitro*, one of the [4Fe–4S] clusters, potentially also the [2Fe–2S] cluster, are highly susceptible to degradation by molecular oxygen. Intriguingly, genetic studies in various eukaryotes have also indicated a connection of Nar1 homologs to O_2_ sensitivity during cell growth.^16,50–53^ The human homolog, CIAO3, and Nar1 are selectively essential in high oxygen tensions^51,52^ and interactions with the CIA scaffold and the CTC are influenced by the redox status of the cell.^53,54^ Together with the *in vivo* data from the literature, our data here support a further function of Nar1 as a redox component that regulates the CIA pathway. How this redox function may couple to the proposed function of Nar1 as an [4Fe–4S] trafficking component will be explored in future studies.

Based on the Mössbauer and MS data together, a third binding site for a [2Fe–2S] cluster exists in Nar1. As there are no other obvious conserved Fe/S binding sites (Fig. S1), it is hypothesized that the [2Fe–2S] cluster bound to Nar1 may occupy the cavity in which the [2Fe]_H_ cluster resides in [FeFe]- hydrogenases (Fig. 5). The conserved Cys_416_ ligand analogous to the bridging cysteine in hydrogenases could anchor the [2Fe–2S] cluster in the cavity (Fig. 1 and 5); however, the homology model provides no clear indication into how the remaining coordination environment could be fulfilled. It may be possible that additional sulphide/disulphide (HS^−^/HSS^−^) species^56,57^ bind to the [2Fe–2S] cluster that at the moment cannot be distinguished from protein disulphides. Whereas the evidence for a [2Fe–2S] cluster in the cavity of reconstituted protein can be drawn from Mössbauer and MS spectra, whether the [2Fe–2S] cluster also possibly resides in the cavity in the as-isolated state cannot be as readily traced at this time. Yet, the lack of a [2Fe–2S]^+^ signal by Mössbauer for the reduced as-isolated sample would suggest that the [2Fe–2S] cluster in the oxidized sample did not reside in a 4-Cys coordination environment in sites 1 or site 2, as this scenario would be expected to give a stable ferredoxin-like [2Fe–2S]^+^ spectrum. Furthermore, the destruction of the [2Fe–2S] cluster observed by Mössbauer for both the as-isolated and reconstituted protein suggests that the [2Fe–2S] cluster is labile in both states and hence resides in the same binding site (Fig. 5). The lability of the [2Fe–2S] cluster was also evidenced by the partial breakdown of [2Fe–2S] species by MS and the apparent exchange of ^56/57^Fe isotopes in the ^56/57^Fe labelling experiment. In further support of the cavity of Nar1 accommodating cofactors, hydrogenase activity has been observed in Nar1 homologs that have been isolated from *E. coli* grown under anaerobic conditions.^58^ Further detailed spectroscopic and structure determination studies are underway to understand the binding of Fe/S clusters to Nar1.

## Conclusions

In this study, the binding of multiple Fe/S clusters to the essential eukaryotic protein Nar1, including an unprecedented [2Fe–2S] cluster, has been demonstrated by spectroscopic and spectrometric methods. It is predicted that a [2Fe–2S] cluster can take the place of the unique [2Fe]_H_ cofactor found in [FeFe]-hydrogenases. Instead of hydrogen metabolism, our biochemical results support the hypothesis that the Fe/S clusters in Nar1 homologs are important in oxygen sensing as has been suggested by previous physiological studies, in addition to the role as a CIA component influencing the trafficking of [4Fe–4S] clusters to target proteins. While it remains unclear if a [2Fe–2S] cluster is physiologically relevant in yeast and other eukaryotes, this work provides evidence for Nar1 being a further cytosolic Fe/S protein capable of binding [2Fe–2S] clusters that can interact with the CIA targeting complex,^11,13^ as observed for the protein Apd1.^14,29,59^ Interestingly, Fe/S cluster binding to Apd1 is dependent on Nar1.^29^ How Nar1 may facilitate the transfer of either [4Fe–4S] or [2Fe–2S] clusters to target proteins is a question that warrants further studies. Our study now allows for the production of multiple Fe/S cluster-loaded forms of Nar1, which can be utilized in *in vitro* reconstitution assays to probe the molecular functions of the CIA pathway.

## Author contributions

J. J. B. and J. C. C., conceptualization; J. J. B., L. K., J. C. C., J. O., and J. C. S. investigation; J. J. B., L. K., J. C. C., J. O., M. H., N. E. L. B., V. S., and M. K. formal analysis; J. J. B., J. C. C., N. E. L. B., V. S., M. K. validation; J. J. B., L. K., and J. C. C. visualization; J. J. B. writing – original draft; J. J. B., L. K., J. C. C., N. E. L. B., V. S., and M. K. writing – review & editing; all authors approved the manuscript; J. J. B. project administration; J. J. B., J. C. C., N. E. L. B., V. K., and M. K. funding acquisition.

## Conflicts of interest

There are no conflicts to declare.

## Supplementary Material

SC-017-D5SC04860E-s001

## Data Availability

The data supporting this article can be found within the text and within the supplementary information (SI). Supplementary information: SI Fig. S1–11 and Tables S1–4, SI Methods. See DOI: https://doi.org/10.1039/d5sc04860e.
